# Age and environmental factors predict psychological symptoms in adolescent refugees during the initial post-resettlement phase

**DOI:** 10.1186/s13034-022-00538-y

**Published:** 2022-12-20

**Authors:** Debbie C. Hocking, Suresh Sundram

**Affiliations:** 1 Cabrini Outreach, 183 Wattletree Road, Malvern, VIC 3144 Australia; 2 Department of Psychiatry, School of Clinical Sciences, Faculty of Medicine, Nursing and Health Sciences, Monash University, Wellington Rd, Clayton, VIC 3800 Australia; 3 Mental Health Program, Monash Health, 246 Clayton Road, Clayton, VIC 3168 Australia

**Keywords:** Adolescents, Resettled refugees, Risk factors, Posttraumatic symptoms, Depression symptoms, Displacement

## Abstract

**Background:**

Adolescent refugees are at high risk of developing mental disorders but are often not recognised early. This pilot study aimed to identify early putative risk factors associated with psychological symptoms in newly resettled refugee youth at potential risk of subsequently developing mental disorders.

**Methods:**

Newly resettled adolescent refugees were recruited through English language schools in Melbourne, Australia. Participants were assessed with the MINI-Kid, Achenbach Youth Self-Report and Reaction of Adolescents to Traumatic Stress scale. Parents completed a mental health screening separately. Linear regression models were used to identify predictive factors associated with symptom ratings.

**Results:**

Seventy-eight, ostensibly well, refugee adolescents (*mean age* = 15.0 ± 1.6 years) resettled in Australia for 6.1 ± 4.2 months were assessed. Levels of anxiety, depression and post-traumatic stress symptoms were considerably lower than in mainstream population data. Prior displacement was a key determinant of symptomatology. Transitory displacement, irrespective of duration, was associated with elevated scores for depression (*t *(47) = -4.05, *p* < 0.0001), avoidance/numbing (*U* = 466, *p* < .05) and total trauma (*U* = 506, *p* < .05) symptoms. Older age was a unique predictor of depression (*F* (1,74) = 8.98, *p* < .01), internalising (*F*(1,74) = 6.28, *p* < .05) and total (*F*(1,74) = 4.10, *p* < .05) symptoms, whilst parental depression symptoms (*t* = 2.01, *p* < 0.05), displacement (*t* = 3.35, *p* < 0.01) and, expectedly, trauma exposure (*t* = 3.94, *p* < 0.001) were unique predictors of post-traumatic stress symptoms.

**Conclusions:**

Displaced status, older age, and parental symptoms predicted psychological symptoms in adolescent refugees in an initial relatively asymptomatic post-resettlement phase. The early recognition of at-risk refugee youth may provide an opportunity for preventative mental health interventions.

**Supplementary Information:**

The online version contains supplementary material available at 10.1186/s13034-022-00538-y.

## Introduction

Currently there is an unprecedented number of forcibly displaced people globally, including 27 million refugees. Whilst those under 18 years of age account for 30% of the world’s population, 41% of all forcibly displaced people are children [1].

Forced migration increases a young person’s risk of developing a mental disorder [2] with meta-analyses finding mental disorders in forced migrant youth to be many-fold higher than the general population in Western host countries [3]. Moreover, adolescent refugees also appear to have higher rates of mental disorders than adults [2] and may be more vulnerable than younger children [4]. This may plausibly be due to the coalescence of exposure to potentially traumatic pre-, peri- and post-migration factors [5] occurring in adolescence, a particularly vulnerable period for the development of psychopathology [6].

In mainstream adolescent populations, symptom load has been identified as a key risk factor for the onset of anxiety and mood disorders, and early sub-threshold symptoms—often mediated by stressful life events—have been shown to increase the risk of developing a mental disorder [7]. However, no work has been done identifying risk factors for psychological symptoms in newly resettled adolescent refugees as possible predictors of subsequent mental disorders.

A characteristic of the longitudinal mental health profile of resettled refugee youth appears to be a temporal variation in symptom and disorder intensity and prevalence [8]. This may include an initial post-resettlement “honeymoon” phase whereby mental symptoms and psychological distress are not experienced, or are minimised, due to the excitement or euphoria of safe arrival and the activity of resettlement [9]. The duration may variably persist, and mental symptoms and disorders may only emerge subsequently in the context of post-migration stress [8]. This may result in the immediate post-resettlement phase of high false negative rates for mental health screening, in turn, leading to delayed or missed intervention opportunities.

It is plausible that psychological symptoms or distress manifesting in otherwise apparently mentally well refugee adolescents in the initial post-resettlement phase may be indicative of heightened risk for subsequent mental disorders. Hence, this study sought to determine the presence of psychological symptoms in refugee adolescents in the initial post-resettlement phase and to identify associated factors. We considered adolescent refugees to be individuals aged between 12 and 17 years whose primary carers had been granted permanent protection visas by the Australian government on humanitarian grounds.

## Methods

### Procedure

Consecutive sampling of refugee adolescents was conducted from May to December 2019 at two campuses of the Western English Language School in Melbourne, Australia. Eligible participants, aged 12–17, were newly resettled refugees (≤ 12 months). Adolescents who were either diagnosed with a mental disorder or had received mental health treatment since arriving in Australia, were excluded. The study comprised three parts: (1) mental health screening of the adolescent; (2) structured psychiatric interview with the adolescent; and (3) interview with the parent (or legal guardian).

The Welfare Co-ordinator at each school campus contacted eligible families to explain the research and seek verbal consent for participation in the study. Written consent from both the adolescent and their parent/legal guardian was obtained prior to the adolescent being screened. After screening by the school welfare officer, the adolescent undertook an interview protocol with the researcher. When possible, the adolescent’s parent (or guardian) then undertook the parent interview protocol with the researcher.

The respective protocols are described below. All screening and interviews were conducted at the adolescent’s school, and interviews with parents and adolescents were undertaken separately. English language interpreters were engaged as required, and adolescent participants were compensated for their time (AUD25) at the completion of the post-screening interview.

### Materials

Language translations of the self-report measures were utilised for the adolescent and parent protocols when available. Alternatively, these were completed in situ with the assistance of an interpreter.

#### Adolescent interview protocol

The interview protocol collected socio-demographic information and clinical information relevant to mental health. In addition to completing self-report measures each participant undertook a semi-structured psychiatric interview (MINI-Kid 7.0.2 for ICD-10 and DSM-5) [10] to identify unrecognised mental disorders.

The Achenbach Youth Self-Report (YSR) [11] is a 112-item self-report measure of psychological and behavioural disturbance for 11–18 year olds which comprises eight ‘syndrome’ and seven ‘DSM-oriented’ scales [12]. The Reactions of Adolescents to Traumatic Stress (RATS) scale [13] is a 22-item self-report measure which assesses trauma symptoms. The RATS comprises three subscales, corresponding to DSM-IV/ICD-10 posttraumatic stress disorder (PTSD) symptom clusters: intrusions, avoidance/numbing and hyperarousal.

The Strengths and Difficulties Questionnaire (SDQ) [14] is a 25-item measure of psychosocial difficulties and emotional distress in children and youth. It has been used extensively in Western and non-Western populations [15]. Both the 11–17 year old self-report form and the parent form were administered. The present paper focuses exclusively on data from the clinical scales (i.e., YSR and RATS).

The RATS was developed for refugee adolescent populations, and the YSR and SDQ have been found to be valid measures of psychosocial-emotional and behavioural symptoms across a broad range of cultures [16]. All three scales have good internal consistency (RATS, 0.88; YSR, 0.95; SDQ, 0.80) [17].

#### Parent interview protocol

Parents were screened for likely PTSD and depression using the Screening Tool for Asylum seeker and Refugee Mental Health (STAR-MH) [18]. PTSD and depression/anxiety symptoms were assessed using gold standard self-report scales—the Harvard Trauma Questionnaire (HTQ-R) and Hopkins Symptom Checklist (HSCL-25) [19]. Parental caseness for likely PTSD was determined by agreement between the STAR-MH screening result and a cut-score of 2.0 on the HTQ and HSCL, as per the utilisation guidelines for these measures [19].

### Data analysis

#### Treatment of data

The following demographic variables were computed as grouped data to facilitate analyses: country of origin; ethnicity; language; and pre-migration displacement. The term ‘displaced’ is used henceforth to denote transient, or interim displacement.

Given the number of countries represented (n = 11), Country of origin was grouped into Thailand (n = 33), Iraq (n = 16), Myanmar (n = 8), DRC (n = 7), and Other (n = 14). Nine ethnicities were represented, with ethnicity being grouped into Karen (n = 30; Thailand), Assyrian (n = 16; Iraq), Congolese (n = 10; DRC) and Other (n = 22). Language was divided into four groups: Karen (n = 30), Arabic (n = 21), Swahili (n = 10) and Other (n = 17). Skewed group counts for religion (78% of sample being Christian) did not render this a meaningful variable for further analysis.

Trauma exposure (PTSD Criterion A) was elicited in response to the MINI-Kid PTSD module. PTSD Criterion A and number of traumatic events experienced (i.e., categories of traumatic events) were used as categorical and dimensional measures of trauma exposure, respectively.

Given the potential role of transient displacement in the pre-migration context, the sample was divided into those with and without an experience of displacement. Those resettled in Australia from their country of origin or born in a refugee camp were allocated to the Non-displaced group (n = 46); those who spent time in a transit country or refugee camp but were not born in a camp were designated to the Displaced group (n = 32).

Prior to conducting linear and hierarchical regressions, analyses and data inspections were performed to identify violations of linearity, homoscedasticity, independence and normality. Collinearity diagnostics were performed on relevant independent variables. Conservative parameters were applied to address multicollinearity: variance inflation factor (VIF) > 2.5 [20], tolerance < 0.2 and r ≥ 0.70 [21]. Mahalanobis distance metric was applied to all regression equations, and outliers were removed as necessary. Variables contributing to collinearity were iteratively removed from the remaining analyses, which were then recalculated until the final model met the assumption criteria.

#### Statistical analyses

Analyses were run separately for each of the clinical outcome measures: YSR, RATS, and their respective subscales. In the first instance, correlations were conducted to ascertain bivariate relationships between adolescent symptom scores and both socio-demographic and parent clinical variables. Further analyses were undertaken with variables found to be significant at the p < 0.05 level.

Pearson product-moment and Spearman’s rank correlations were employed for parametric and non-parametric data, respectively. *T*-tests and analyses of variance were conducted to determine group differences for symptom measures. Mann–Whitney U tests and Kruskal–Wallis tests were applied to non-normally distributed dimensional data. Chi Square tests were employed for bivariate analyses of categorical variables.

Multiple linear and hierarchal regression analyses were conducted to determine socio-demographic and clinical predictors of symptom scales. Statistical analyses were conducted using the Statistical Package for the Social Sciences (SPSS) for Windows v.22. An alpha of 95% was applied throughout. False discovery rate (FDR) post hoc tests were applied to multiple comparison analyses.

## Results

### Participants

**Fig. 1:**
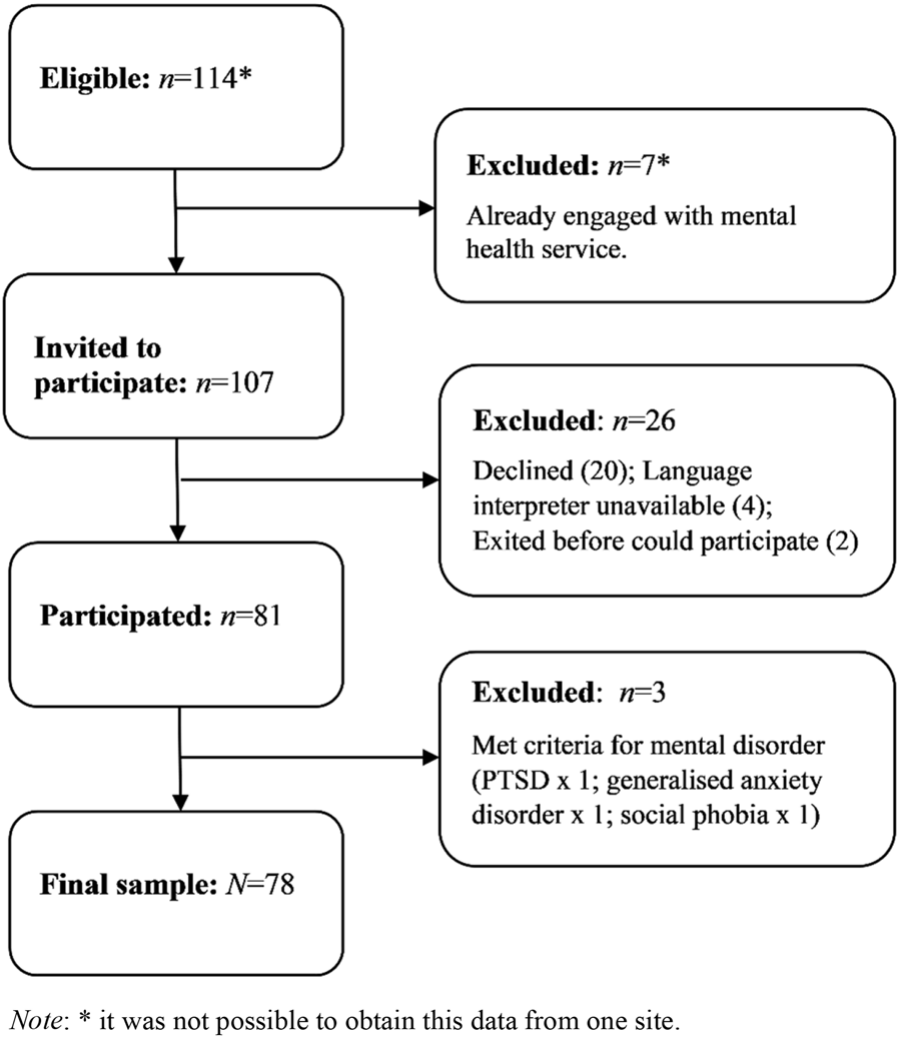
Consort diagram of sample.

The participant response rate was 81.3%. Recruitment flow is presented in Fig. 1. Three adolescent participants were identified by the MINI-Kid as having a mental disorder and were removed from subsequent analyses.

Seventy-eight newly arrived refugees aged 12–17 years from 53 families comprised the final sample. Additional file 1: Table S1 presents the key socio-demographic characteristics of the sample.

Participants were resettled from 11 countries of origin, with almost two-thirds being collectively from Thailand and Iraq.

The average time resettled in Australia was six months. Of those who had lived in a refugee camp approximately three-quarters were born in a camp and the remainder had a mean camp duration of seven years. Approximately one-quarter had spent time in a transit country (urban setting), with the median duration being four years. The remainder were eleven adolescents resettled directly from their country of origin, 10 (90%) of whom were family-sponsored refugees.

Family composition of the adolescent participants and relevant socio-demographic data of participating parents is shown in Additional file 1: Table S1. Almost all participants (n = 74; 94.9%) had at least one parent in Australia, whether emigrating with a parent, or having a parent sponsor their passage. Fifteen (93.8%) of the sixteen single-parent households were female-headed and, in six (37.5%) cases, were the result of the death of the other parent.

Parent interviews were conducted for 88.5% of the adolescent sample with the majority being mothers, and requiring an English language interpreter.

### Trauma experience

Approximately one third (35.9%) of the adolescents reported having experienced a traumatic event (PTSD criterion A). Of these, 82% reported exposure to only one event. The most frequently cited trauma categories were: witnessing violence to others (8); disappearance, death or injury of loved ones (8); and exposure to a natural disaster (fire or flood; 7).

### Correlations and comparisons

The scores for each of the YSR and RATS scales are presented in Table 1.

**Table 1:** Mean and median scores for symptom scales and subscales

YSR Scale (Score range)	Mean (SD)	Md (IQR)	Zeros—*n (%)*
Syndrome scales			
Anxious/depressed (0–26)	2.64 (2.20)	2 (1–4)	13 (16.7)
Withdrawn/depressed (0–16)	1.79 (1.90)	1 (0–3)	27 (34.6)
Somatic complaints (0–20)	1.41 (2.03)	0 (0–2.25)	41 (52.6)
Social problems (0–22)	1.64 (1.64)	1 (0–3)	26 (33.3)
Thought problems (0–24)	0.97 (1.47)	0 (0–2)	44 (56.4)
Attention problems (0–18)	2.29 (2.35)	2 (0–3)	22 (28.2)
Rule-breaking behaviour (0–30)	1.26 (1.55)	1 (0–2)	29 (37.2)
Aggressive behaviour (0–34)	2.41 (2.72)	2 (0–4)	25 (32.1)
Internalising problems^a^ (0–62)	5.85 (4.77)	4 (2–9.25)	6 (7.7)
Externalising problems^b^ (0–64)	3.67 (3.82)	3 (1–5)	16 (20.5)
Total problems^c^ (0–224)	**16.35 (12.75)**	**12 (7–22.5)**	**1 (1.3)**
DSM-oriented scales			
Depressive problems (0–26)	1.73 (2.00)	1 (0–3)	30 (38.5)
Anxiety problems (0–18)	1.95 (1.93)	2 (0–3)	24 (30.8)
Somatic problems (0–14)	0.85 (1.43)	0 (0–1)	48 (61.5)
Attention deficit problems (0–14)	2.00 (1.87)	2 (0–3)	23 (29.5)
Oppositional defiance problems (0–10)	1.05 (1.42)	0 (0–2)	43 (55.1)
Conduct problems (0–30)	1.04 (1.66)	0.5 (0–2)	39 (50.0)
Obsessive compulsive problems (0–16)	1.87 (1.72)	2 (0–3)	21 (26.9)
RATS			
Hyperarousal (7–28)	8.09 (1.81)	7 (7–9)	46 (59.0)
Intrusive (6–24)	6.59 (1.17)	6 (6–7)	55 (70.5)
Avoidance/numbing (9–36)	11.82 (3.71)	10.50 (9–13)	34 (43.6)
RATS (PTSD) total (22–88)	**26.50 (5.47)**	**24 (22–29.3)**	**25 (32.1)**

^a^comprises Anxious/Depressed, Withdrawn/Depressed and Somatic Complaints scales

^b^comprises Rule-breaking and Aggressive behaviour

^c^sum of all YSR items

All independent variables were entered into correlation matrices with all outcome measures: 18 YSR scales and 4 RATS scales.

Group comparisons were calculated with all significant variables that permitted such analyses. As shown in Additional file 2: Table S2, only results that remained significant after applying corrections for multiple comparisons are reported.

### YSR

Eleven variables were significantly associated with one or more of the YSR scales. These were: age, sex, country of origin, ethnicity, language, parent pre-arrival occupation, displaced status, trauma exposure (PTSD Criterion A), number of traumatic events, time in Australia, and parent depression score. The socio-demographic factors associated with the YSR subscales are presented in Additional file 2: Table S2.

Older age was a consistent demographic predictor of symptoms and was associated with several of the YSR scales (Table 2). The older age group (15–17 years) scored significantly higher than the younger age group (12–14.9 years) on six symptom scales.

**Table 2:** YSR syndrome and DSM-oriented scales associated with older age

YSR scale	Correlation	Group comparison^a^
Syndrome scales		
Anxious/depressed	*r* = .294, *p* = .009^*^	*t*(74) = -1.81, *p* = 0.074
Withdrawn/depressed	*r* = .292, *p* = .009^*^	*t*(74) = -2.769 *p* = 0.027^*^
Somatic complaints	ρ = .173, *p* = .131	*U* = 501, *Z* = -2.47, *p* = 0.039^*^
Social problems	*r* = .151, *p* = .186	*t*(74) = -1.65, *p* = 0.103
Thought problems	ρ = .251, *p* = .026^*^	*U* = 550, *Z* = -1.96, *p* = 0.051
Attention problems	*r* = .200, *p* = .079	*t*(74) = -1.55, *p* = 0.125
Rule-breaking behaviour	ρ = .071, *p* = .536	*U* = 613, *Z* = -1.17, *p* = 0.241
Aggressive behaviour	ρ = .207, *p* = .069	*U* = 568, *Z* = -1.62, *p* = 0.105
Internalising problems^b^	*r* = .306, *p* = .006^**^	*t*(71) = -3.11, *p* = 0.027^*^
Externalising problems^c^	ρ = .134, *p* = .241	*U* = 599, *Z* = -1.27, *p* = 0.204
Total problems^d^	*r* = .276 *p* = .014^*^	*t*(71) = -2.75, *p* = 0.027^*^
DSM-oriented scales		
Depressive problems	ρ = .366, *p* = .001^**^	*U* = 439, *Z* = -3.02, *p* = 0.009^**^
Anxiety problems	*r* = .215, *p* = .059	*t*(74) = -1.56, *p* = 0.123
Somatic problems	ρ = .232, *p* = .041^*^	*U* = 459, *Z* = -3.12, *p* = 0.009^**^
Attention deficit problems	*r* = .125, *p* = .277	*t*(74) = -0.973, *p* = 0.334
Oppositional defiance problems	ρ = .204, *p* = .074	*U* = 560, *Z* = -1.83, *p* = 0.068
Conduct problems	ρ = .020, *p* = .860	*U* = 687, *Z* = -0.377, *p* = 0.706
Obsessive compulsive problems	*r* = .231, *p* = .042^*^	*t*(74) = -1.30, *p* = 0.198

Statistics in bold are significant after correcting for multiple comparisons. Benjamini–Hochberg Adjusted p values are displayed

^a^older group (15–17 years) > younger group (12–14.9 years)

^b^comprises anxious/depressed, withdrawn/depressed and Somatic Complaints scales

^c^comprises Rule-breaking and Aggressive behaviour

^d^sum of all YSR items

^*^Significant at the 0.05 level

^**^Significant at the 0. 01 level

Those who had been displaced returned a significantly higher score for withdrawn/depressed symptoms (Additional file 2: Table S2), but there was no relationship between displacement time (absolute or as a proportion of age) and symptom scores in this group. In contrast, time spent in the pre-migration setting for the non-displaced group was inversely associated with withdrawn/depressed symptoms (ρ = − 0.350, p = 0.018), social problems (ρ = − 0.441, p = 0.002), attention problems (ρ = − 0.398, p = 0.007), attention deficit symptoms (ρ = − 0.381, p = 0.010), obsessive compulsive symptoms (ρ = − 0.378, p = 0.010), thought problems (ρ = − 0.367, p = 0.013) and total symptoms (ρ = − 0.366, p = 0.013). These associations held for all but thought problems when corrected for age by using time spent in non-displaced settings as a proportion of age.

Time in Australia was positively related to both withdrawn/depressed symptoms and oppositional defiance problems (Additional file 2: Table S2).

### RATS

Seven variables were significantly associated with one or more of the RATS scales. These were: country of origin, ethnicity, displaced status, trauma exposure (PTSD criterion A), number of traumatic events, time in Australia, and parent depression score (Additional file 2: Table S2).

The displaced group scored higher than the non-displaced group on avoidance/numbing and total trauma symptoms despite there being no difference in trauma exposure (PTSD Criterion A) (χ^2^ (1) = 2.84, p = 0.09) or number of traumatic events between the two groups (U = 541, Z = − 1.12, p = 0.26).

Time in Australia was positively related to intrusive symptoms.

### Parent data

Parent data from the three excluded adolescent cases were also removed, resulting in a parent sample of N = 44 for socio-demographic data and subsequent analyses. As with the adolescent participants, parents returned low symptom scores, and only three parents met caseness for likely PTSD and/or major depressive disorder.

Parental symptoms were positively associated with several adolescent symptom scale scores (See Additional file 2: Table S2). However, there was a gender effect, with maternal and paternal symptom scales correlating differentially for several adolescent mental health indices.

Higher maternal depression symptoms were related to increased thought problems (ρ = 0.367, p = 0.02), avoidance/numbing (ρ = 0.444, p = 0.004), hyperarousal (ρ = 0.313, p = 0.049) and total trauma (ρ = 0.317, p = 0.046) symptoms in their adolescent children. Maternal PTSD symptoms were related to avoidance/numbing symptoms (ρ = 0.340, p = 0.032), and maternal anxiety symptoms were associated with avoidance/numbing symptoms (ρ = 0.323, p = 0.042).

In contrast, only two of the paternal mental health scales were related to adolescent mental health indices: high paternal PTSD scores were associated with decreased DSM conduct problems (ρ = − 0.396, p = 0.041), and high paternal anxiety scores were associated with decreased social problems (ρ = − 0.424, p = 0.028).

Parental symptom scores differed by displacement. As with the adolescents, the displaced parent group returned higher depression scores than the non-displaced group (U = 296, Z = − 2.70, p = 0.021).

### Regressions

#### YSR

Grouped variables language, country of origin and ethnicity were highly inter-correlated. To address collinearity, the latter was retained over the former two variables when more than one was a significant correlate. This was decided on the basis that ethnicity was a stronger predictor of symptom scores on most scales. Similarly, number of traumatic events was retained over PTSD Criterion A.

Linear regressions were performed on each of the 18 YSR scales with the associated predictor variables of each (see Additional file 2: Table S2) entered simultaneously.

The models for the conduct problems and both somatic scales were not feasible because the somatic complaints scale produced no significant predictors, and the DSM-oriented somatic symptoms and conduct problems scales failed linearity tests. The model for the aggressive behaviour scale did not reach significance. Results for the remaining YSR scales are presented in Additional file 3: Table S3.

To further elucidate the role of age in symptom scores whilst controlling for the effects of trauma and displacement, hierarchical regressions were performed on the YSR scales with trauma symptoms and displaced status (Step 1) and age (Step 2) entered as predictor variables (see Table 3). After controlling for displacement and trauma symptoms, age contributed a small but significant level of variance for depressive problems (8.1%), and anxious/depressed (4.5%), withdrawn/depressed (3.0%), internalising (4.3%) and total (3.2%) symptoms.

**Table 3:** Contribution of age after controlling for trauma symptoms and displacement, for YSR Syndrome and DSM-oriented scales

YSR scale	Block 1^a b^		Block 2^c^	
	∆R^2^	Change statistics	∆R^2^	Change statistics
Syndrome scales				
Anxious/depressed	0.369	*F*(2,75) = 21.91^***^	**0.045**	*F*(1,74) = 5.73, *p* = 0.02^*^
Withdrawn/depressed	0.464	*F*(2,75) = 32.47^***^	**0.030**	*F*(1,74) = 4.40, *p* = 0.04^*^
Somatic complaints	0.175	*F*(2,75) = 7.97^**^	0.009	*F*(1,74) = 0.81 *p* = 0.37
Social problems	0.215	*F*(2,75) = 10.25^***^	0.008	*F*(1,74) = 0.76, *p* = 0.39
Thought problems	0.323	*F*(2,75) = 17.88^***^	0.014	*F*(1,74) = 1.52, *p* = 0.22
Attention problems	0.280	*F*(2,75) = 14.59^***^	0.015	*F*(1,74) = 1.59, *p* = 0.21
Rule-breaking behaviour	0.044	*F*(2,75) = 1.73	0.000	*F*(1,74) = 0.01, *p* = 0.99
Aggressive behaviour	0.235	*F*(2,75) = 11.50^***^	0.014	*F*(1,74) = 1.42, *p* = 0.24
Internalising problems	0.450	*F*(2,75) = 30.73^***^	**0.043**	*F*(1,74) = 6.27, *p* = 0.02^*^
Externalising problem	0.184	*F*(2,75) = 8.46^***^	0.007	*F*(1,74) = 0.65, *p* = 0.42
Total problems	0.396	*F*(2,75) = 24.56^***^	**0.032**	*F*(1,74) = 4.10, *p* = 0.046^*^
DSM-oriented Scales				
Depressive problems	0.251	*F*(2,75) = 12.58^***^	**0.081**	*F*(1,74) = 8.98, *p* = .004^**^
Anxiety problems	0.287	*F*(2,75) = 15.12^***^	0.020	*F*(1,74) = 2..16, *p* = 0.15
Somatic problems	0.124	*F*(2,75) = 5.32^**^	0.005	*F*(1,74) = 0.43, *p* = 0.52
Attention deficit problems	0.158	*F*(2,75) = 7.03^**^	0.003	*F*(1,74) = 0.30, *p* = 0.59
Oppositional defiance problems	0.183	*F*(2,75) = 8.40^***^	0.013	*F*(1,74) = 1.18, *p* = 0.28
Conduct problems	0.024	*F*(2,75) = 0.90, *p* = 0.41	0.000	*F*(1,74) = .006, *p* = 0.94
Obsessive compulsive problems	0.377	*F*(2,75) = 22.68^***^	0.020	*F*(1,74) = 2.47, *p* = 0.12

Statistics in bold are significant after correcting for multiple comparisons. Benjamini-Hochberg Adjusted p values are displayed

^a^RATS total (trauma) score

^b^Displaced/not displaced

^c^Age

^*^Significant at the 0.05 level

^**^Significant at the 0.01 level

^***^Significant at the 0.001 level

#### RATS

The same procedure as the YSR regression analyses was followed for the seven RATS predictor variables.

All relevant socio-demographic variables were entered simultaneously into regression analyses for each of the four trauma scales. The intrusive scale failed the linearity and homoscedasticity tests, so was excluded from further analyses. One outlier was removed from the analyses of the avoidance/numbing and RATS Total scales to generate the final models. Additional file 3: Table S3 presents the regression results. The final models explained 37.5% and 33.8% of variance in avoidance/numbing and total trauma symptom scores, respectively. The significant predictors of trauma symptoms were number of traumatic events, displaced status, and parental depression symptoms.

Hierarchical linear regressions were then performed on each of four RATS scales (see Table 4). Number of traumatic events was entered at Step 1, parental depression symptoms were entered at Step 2, and displaced status was entered as Step 3 in the model. Displaced status was a significant predictor of avoidance symptoms after controlling for trauma exposure and parental depression symptoms.

**Table 4:** Displacement as predictor of trauma symptoms scores after controlling for number of traumatic events and parental depression symptoms

Scale	Block 1^a^	Block 2^b^		Block 3^c^		
	∆R^2^	Change statistics	∆R^2^	Change statistics	∆R^2^	Change statistics
Avoidance/numbing	0.294	*F*(1,62) = 25.76^**^	0.032	*F*(1,61) = 2.93	0.073	*F*(1,60) = 7.29, *p* = .009*
RATS (PTSD) total	0.252	*F*(1,62) = 20.89^**^	0.081	*F*(1,61) = 7.46**	0.036	*F*(1,60) = 3.39, *p* = .071

^a^Number of traumatic events

^b^Parental depression symptoms

^c^Final model after entering displaced status

^*^Significant at the 0.01 level

^**^Significant at the 0.001 level

## Discussion

This study identified for the first time, the initial post-resettlement phase for adolescent refugees as psychologically quiescent. It went on to demonstrate age, displacement, parental depression and exposure to trauma as risk factors for psychological symptoms in the initial post-resettlement phase. These novel data complement previous studies that identified comparable risk factors for established mental illness in resettled refugee youth including sex, age, ethnicity, exposure to violence, family factors, parental factors, time since displacement, and number of relocations within the host country [22]

The sample of non-clinical, ostensibly healthy adolescents and the early time-frame—within the first 12 months of resettlement—were an intentional focus of the study in order to investigate antecedent factors associated with psychological symptoms in the initial post-resettlement “honeymoon” phase ascribed to arrival in a safe haven environment [9, 23]. The low psychological symptom scores confirmed, for the first time, that this honeymoon phase was present in an adolescent refugee sample with scores substantially lower on the YSR than in host populations. A mean score of 35.3 for the 98-item YSR was found across 24 countries [24], whilst in this study the mean score was 15.9 using the 112-item YSR. Similarly, RATS scores on the 90^th^ percentile in our sample equated with that of the 10^th^ percentile in a large Dutch sample of unaccompanied refugee minors [17].

### Displacement

The findings of this study are the first to our knowledge, to quantify the role of displacement in mental health of adolescent refugees and presents the novel finding of both adolescent and parental psychological symptoms being related to pre-resettlement displacement. A critical determinant of resilience in refugee adolescents is the socio-ecological context [25] and, hence, the pre-migration milieu such as residing in a stable refugee camp may mitigate or prevent psychopathology through provision of relative security, stability, and predictability.

In contrast to previous findings of an inverse dose–response relationship between psychopathology and more than two years of displacement [3], we found no correlative relationship. Instead, it was the quality of the displacement experience that was predictive. Specifically, transient displacement in a transit country or environment was associated with psychological symptoms whereas no experience of a transit environment or long-term displacement in a stable environment were comparatively protective. Whilst this finding has emerged for adult refugees [26], we now extend this to include refugee youth.

Adolescents who had not been displaced returned lower depression and trauma symptoms than those who had been displaced. Furthermore, whilst there was no correlation with time for those who had been displaced, scores for several symptom measures were inversely related to time for the non-displaced group as a proportion of participant age.

Disparities in trauma symptom scores between the displaced and non-displaced groups emerged despite no significant differences in pre-migration trauma exposure. This indicates that protective factors in not being displaced and/or risk factors in displacement may mitigate or exacerbate the psychological impact of any prior trauma exposure.

Three-quarters of those with a refugee camp experience had been born in camp and therefore did not have an experience of pre-resettlement displacement. A study set in a warzone of the former Yugoslavia [27] found nonviolent trauma such as relocation and deprivation to be associated with higher anxiety and depression symptoms in displaced children compared to those not displaced. Hence, trauma related to displacement, relocation and deprivation may cumulatively contribute to psychological symptoms in refugee youth.

A stable and financially sustainable living situation for refugee adults has been proposed to be the most important factor in reducing the risk of mental disorder [28]. The present findings build upon this, indicating that minimising relocations or residing in a comparatively safe and stable refugee camp, may be protective for refugee youth. In contrast, transient residence in transit countries may be a risk factor for psychological symptoms. Hence, adolescents with transit-country journeys may be more vulnerable than those resettled directly from relatively well-resourced, organised and stable camps.

The provision of organised displacement or assisted relocation to a ‘good enough’ setting with a sense of community has been found to both mitigate mental distress and promote psychosocial wellbeing [29]. Accordingly, lower levels of psychopathology have been reported on the basis of a camp’s infrastructure, community integration, and level of violence [30]. For example, a study conducted in Pakistani refugee camps found a significantly higher ratio of children in longer established camps to be happier in their setting compared to those in newer camps, independent of the desire to return to their homeland [31]. Similar to the present study, this referenced camp population did not have any direct experience of war-related violence or of asylum-seeking—two significant risk factors for psychopathology. To our knowledge, our study is the first that extends the psychologically protective milieu of a stable pre-resettlement environment into the post-resettlement phase.

Another plausible explanation for increased symptoms for those in the displaced compared to the non-displaced group is the experience of life in transit [32]. The strain imposed by this may be akin to that of asylum-seekers in host countries, for whom the admixture of prolonged uncertainty, unemployment or insecure employment, straitened financial circumstances, lack of access to health care, and discrimination, contribute to stress. Post-migration factors are known to contribute equivalent or greater levels of psychological symptoms as pre- and peri-migration trauma [33], and can predict psychological symptom severity independent of pre-migration factors [34]. Given these situational equivalencies, it is conceivable that displacement erodes resilience in a similar way.

### Age

The incidence of mood disorders rises sharply during adolescence [35], but this trend appears more equivocal in refugee youth [22]. Notwithstanding positive findings of increased depressive symptoms for adolescent refugees in relation to age, there remains the challenge of disaggregating age from confounding risk factors, such as the accumulation of traumatic events [22], accompaniment status [36], and age of emigration [37].

We found that the severity of depression symptoms was age-dependent, with older adolescents being more vulnerable. Two previous studies with adolescent refugees newly resettled in high income countries found that older age predicted increased self-reported depression symptoms [36, 38]. However, Derluyn & Broekaert [38] noted the potentially confounding relationship between older age and number of traumatic events. In our sample, once trauma symptoms were controlled for, age maintained its significance, albeit as a weak predictor of depression symptoms. Bean et al. [36] reported a relationship between age and depression symptoms after controlling for trauma exposure in a large Dutch sample of unaccompanied refugee adolescents and, significantly, we now extend this to youth in family units.

Hence, this study builds upon previous research to highlight the role of older age as a predictor of depression symptoms independent of trauma exposure. What the present findings do not elucidate however, is the relationship between mood symptoms and age in relation to age at emigration. It is possible that older resettled adolescents experience poorer psychological adjustment due to leaving behind friendship networks at a time when peers are critical to psychosocial development [39]. Loss and disruption may have stronger influences on older refugee youth [40]. The notion of existential security—a sense of order, stability, routine and predictability—may also have particular salience for this group [41] as they develop their self-identity within the resettlement context.

Our findings are consistent with epidemiological findings of an Australian population survey which identified a spike in depressive disorder in adolescents (12–17 years) in comparison to children (4–11 years), with particularly high rates for older adolescents (i.e., 16–17 vs 11–15) [42].

The effect of age on heightened depression symptoms in refugee youth requires further investigation, including whether elevated symptoms place them at greater risk than the general population of developing mood disorders.

### Parental factors

Maternal symptoms of anxiety, depression and PTSD were positively associated with psychopathology in the adolescents, aligning with previous research [43]. Our findings identified parental depression symptoms as a unique predictor of adolescent trauma symptoms, and were also related to increased oppositional symptoms.

Parental mental ill-health has potential clinical significance for adolescents via several possible mechanisms. Avoidance or numbing symptoms may result in parents’ inability to recognise psychopathology in their adolescent children [44]—particularly in relation to internalising symptoms [45]. This lack of recognition may be a contributor to the under-utilisation of mental health treatment services in vulnerable refugee youth who may need help [46]. Conversely, good parental mental health is a protective factor for adolescents [25].

### Time since resettlement

Whilst overall symptoms were low in this ostensibly healthy community population, we found a positive association with time since resettlement and intrusive trauma symptoms, depression symptoms and oppositional behaviour symptoms, indicating these symptoms emerged or intensified following resettlement. This concurs with Bean and colleagues [36], who found that length of residence predicted higher internalising, externalising and trauma symptoms in unaccompanied adolescent refugees, the majority of whom had lived in the Netherlands for no more than 18 months.

Whilst the designated resettlement timeframe in our study was confined to the first 12 months, baseline psychopathology may be the strongest predictor of longer term psychopathology for both internalising and externalising symptoms [47, 48]. However, for at least a proportion of refugee adolescents, there is evidence to suggest that some symptoms may begin to wane at around the 5-year mark, whilst overall maintaining high rates of symptomatology as a population [49].

The possible influence of the resettlement timeframe in the emergence of mental health problems for adolescent refugees warrants further investigation due to its clinical implications. It also underscores the importance of mental health screening for refugee youth early in their resettlement trajectory.

### Strengths and limitations

This study is novel in that it investigated the first 12 months of resettlement for apparently well refugee adolescents and incorporated parental psychopathology, which few studies have done [3]. However, there are some limitations which impact the findings. The cross-sectional design cannot imply causation and emphasises the need for a follow-up study to elucidate some of the explanatory constructs proffered. Furthermore, data was not collected specifically on family violence as a risk factor for psychological symptoms [50]. Secondly, whilst all enrolled students who met criteria were invited into the study, random sampling of schools was not possible. Thirdly, it cannot be discounted that those who declined to participate differed from the sampled population. Fourthly, the small sample size constrained the power of the study despite the significant findings. Finally, whilst documented trauma exposure was approximately 36% of the population, our sample was drawn from the approximately 100,000 refugees selected for resettlement in Australia. Hence our participants comprised an unselected sample of the entire refugee population.

### Future research and clinical implications

The psychologically quiescent initial post-resettlement period contrasts with later manifestations of psychological and psychiatric disorder in adolescent refugees. Little is known about the precise timing and the mechanisms for transition into disorder. Therefore it is imperative to fully characterise the transitional phase to develop an evidence based set of interventions that can prevent, delay and/or ameliorate the emergence of psychological and psychiatric disorders in adolescent refugees.

Our risk factors of older age and parental psychological symptoms align with previously identified risk factors for later disorder at the level of individual, family and community, such as pre-migration trauma exposure, parental exposure to pre-migration violence, and post-migration discrimination [22]. Our findings suggests that the antecedents for psychological and psychiatric illness are operating even in a context of apparent wellness, and we can add the newly identified risk factor of pre-migration displacement.

Pre-migration risk factors can be identified at time of entry to enable closer monitoring for these adolescents. Addressing post-migration factors for those with strong pre-migration risk factors of trauma and displacement would likely ameliorate emergent psychological symptoms and reduce risk of developing mental disorders. Non-clinical interventions that emphasise strength-based approaches to build resilience and facilitate positive peer relationships may be a first step by which to promote social inclusion, and mentoring programs for new arrivals may be one such approach [51]. This may reduce social isolation and assist with adapting to a new culture and sociocultural identity, which may be especially important for older adolescents. Community based programs to promote inclusivity, diversity and to reduce racism, especially school-based programs may hold promise.

## Conclusion

This study examined factors associated with psychological symptoms in ostensibly well refugee adolescents in the initial post-resettlement phase—a time when such symptoms are conceptualised to be low. Thus, this study provides an important nexus in understanding the trajectory of the genesis of mental illness in adolescent refugees between studies of currently displaced contexts and longer settled cohorts. It identified risk factors, specifically age, trauma exposure and parental depressive symptoms that have continuity as risk factors for diagnosed mental illness in longer settled cohorts. However, in addition, it identified for the first time, that the displacement context was associated with psychological symptoms.

The findings demonstrated an increased vulnerability of older newly resettled refugee adolescents to higher depression symptom burden, and the putative protective effect of long-term residence in comparatively safe and established refugee camps transitioning into the initial post-resettlement phase. However, this protective factor may dissipate over time, particularly with increased exposure to stressors of the post-resettlement environment. Older age predicted internalising and mood symptoms after controlling for trauma symptoms and pre-migration setting; and parental mental symptoms predicted oppositional defiance problems, and anxiety and trauma symptoms. Hence, adolescence, displacement and parental mental health may converge to induce or exacerbate mood symptoms.

Identifying predictors of increased symptom burden can help identify at-risk adolescents and delineate the stratification of risk to inform targeted, stepped interventions. However, follow up research is needed to determine the fidelity of this model in predicting subsequent onset of mental disorders. Such an outcome would provide an opportunity for truly preventative interventions in adolescent refugees.

## Supplementary Information

**Additional file 1: Table S1.** Socio-demographic characteristics of sample.

**Additional file 2: Table S2.** Socio-demographic predictors of symptoms.

**Additional file 3: Table S3.** Socio-demographic and clinical predictors of YSR and RATS symptom scores.

## Data Availability

For reasons of confidentiality, the data is not available online. Data-related queries should be directed to the authors.

## Abbreviations

RATS: Reactions of adolescents to traumatic stress scale
YSR: Youth self-report
STAR-MH: Screening tool for asylum seeker and refugee mental health
HTQ-R: Harvard trauma questionnaire
HSCL-25: Hopkins symptom checklist
