# Supplementary material for: Age and environmental factors predict psychological symptoms in adolescent refugees during the initial post-resettlement phase
Source: Child Adolesc Psychiatry Ment Health. 2022 Dec 20;16:105. doi: 10.1186/s13034-022-00538-y (PMC9768994; doi:10.1186/s13034-022-00538-y)
Supplement: Supplementary file 1 — Additional file 1: Table S1. Socio-demographic characteristics of sample. [file 13034_2022_538_MOESM1_ESM.pdf]

Table S1 Socio-demographic characteristics of sample

	N (%) [a]		*n*	*M (SD) /Md (IQR)*
**Gender**				
Male	41 (52.6)	Age (yrs)	78	15.0 (1.6)
Female	37 (47.4)	Education (pre-migration yrs)	78	5.4 (2.1)
**Spoke English**		Time in refugee camp (yrs)	46	13.9 (12.0-14.8)
No	75 (96.2)	Born in camp	35	14.2 (1.4)
Yes	3 (3.8)	Not born in camp	11	7.0 (3.6)
**Country**		Time in transit country	21	4.0 (1.3)
Thailand	33 (42.3)	Time since resettlement (mths)	78	6.1 (4.2)
Iraq	16 (20.5)			
Myanmar	8 (10.3)			
Congo (DRC)	7 (9.0)			
Eritrea	5 (6.4)			
Ethiopia	3 (3.8)			
Other	6 (7.7)			
**Ethnicity**				
Karen	30 (38.5)			
Assyrian	16 (20.5)			
Congolese	10 (12.8)			
Chin	8 (10.3)			
Other	9 (11.5)			
**Language**				
Karen	30 (38.5)			
Arabic	21 (26.9)			
Swahili	10 (12.8)			
Burmese	5 (6.4)			
Chin (Hakha)	4 (5.1)			
Other	8 (10.3)			
**Religion**				
Christian	61 (78.2)			
Muslim	7 (9.0)			
Buddhist	8 (10.3)			
Nil	2 (2.6)			
**Visa type**				
Sponsored refugee	41 (53.2)			
Refugee	31 (40.3)			
Women at risk	4 (5.2)			
Partner/Spousal	1 (1.3)			
**Pre-migration setting**				
Refugee camp	46 (59.0)			
Transit country	21 (26.9)			
Neither	11 (14.1)			
**Displaced**				
Yes	32 (41.0)			
No	46 (59.0)			
**Born in refugee camp**				
Yes	35 (44.9)			
No	43 (55.1)			
	***N* (%)** [b]			***Md* (*IQR*)**
Family Composition				
**Parent in Australia**				
Both	58 (74.4)	Number of siblings		3 (2–5)
One	16 (20.5)			
None	4 (5.1)			

**Parent/guardian** [c]	
Mother	40 (58.0)
Father	27 (39.1)
Aunt	2 (2.9)
**Parent spoke English** [c]	
No	66 (95.7)
Yes	3 (4.3)
**Premigration employment**	
No work	21 (28.0)
Unskilled	27 (36.0)
Skilled/Trade/Administration	17 (22.7)
Professional/ Self-employed	10 (13.3)
**Current employment** [c]	
Student (English classes)	35 (53.0)
Carer (English classes)	12 (18.2)
Unskilled	6 (9.1)
Nil work or study yet	13 (19.7)

Note: *a* Total *n*s less than 78 are due to missing data; *b* Refers to valid percentage, excluding missing data; *c* Parent/guardian interviewed, *N*=69
