# Supplementary material for: Age and environmental factors predict psychological symptoms in adolescent refugees during the initial post-resettlement phase
Source: Child Adolesc Psychiatry Ment Health. 2022 Dec 20;16:105. doi: 10.1186/s13034-022-00538-y (PMC9768994; doi:10.1186/s13034-022-00538-y)
Supplement: Supplementary file 2 — Additional file 2: Table S2. Socio-demographic predictors of symptoms. [file 13034_2022_538_MOESM2_ESM.pdf]

Table S2 Socio-demographic predictors of symptoms

YSR Scale	Sex [a]	Age	Origin country [b]	Ethnicity [c]	Language [d]
YSR Syndrome Scales					
Anxious/Depressed		r=.294, p=0.009**			
Withdrawn/Depressed		r=.292, p=0.009**	F(4,73)= 2.86, p=0.029*	F(3,74)= 4.01, p=0.011*	χ^2(3)=12.02, p=.014*
Somatic complaints					
Social problems					F(3,74)= 3.15, p=0.03*
Thought problems		ρ=.251, p=0.026*	χ^2(4)=10.05, p=.04*		
Attention problems					
Rule-breaking behaviour	U=530, Z= -2.40, p=0.016*				
Aggressive behaviour					
Internalising problems		r=.315, p=0.005**			
Externalising problems					
Total problems		r=.285, p=0.012*			
YSR DSM-Oriented Scales					
Depressive problems		ρ=.366, p=0.001**			
Anxiety problems					
Somatic problems		ρ=.232, p=0.041*			
Attention deficit problems				F(3,74)= 3.15, p=0.03*	
Oppositional defiance problems				χ^2(3)=12.89, p=.045*	
Conduct problems	U=540, Z= -2.37, p=0.018*				
Obsessive compulsive problems		r=.231, p=0.042*		F(3,74)= 3.63, p=0.017*	
Trauma scales					
Hyperarousal					
Intrusive					
Avoidance/Numbing				U=479, Z= -2.59, p=0.04*	
RATS Total			U=518, Z= -2.31, p=0.04*	U=497, Z= -2.34, p=0.04*	

Table S2 Socio-demographic predictors of symptoms (Cont.)

YSR Scale	Displaced [e]	Trauma exposure (Criterion A)	No. Traumatic events	Time in Aust.	Parental pre-migration occupation [f]	Parental depression score
YSR Syndrome Scales						
Anxious/Depressed		*t*(41)=-2.63, *p*=0.029*	ρ=.374, *p*=0.001**			
Withdrawn/Depressed	*t*(47)=-4.05, *p*<0.0001***	*t*(43)=-2.59, *p*=0.018*	ρ=.279, *p*=0.017*	*r*=.239, *p*=0.035*		
Somatic complaints						
Social problems						
Thought problems			ρ=.251, *p*=0.032*			ρ=.259, *p*=0.031*
Attention problems						
Rule-breaking behaviour					χ^2(3)=10.5, *p*=.015*	
Aggressive behaviour						
Internalising problems		*t*(47)=-2.91, *p*=0.018*	ρ=.398, *p*<0.0001***			
Externalising problems						
Total problems			ρ=.240, *p*=0.041*			
YSR DSM-Oriented Scales						
Depressive problems			ρ=.278, *p*=0.017*			
Anxiety problems			ρ=.241 *p*=0.04*			
Somatic problems						
Attention deficit problems						
Oppositional defiance problems				ρ=.233, *p*=0.04*		ρ=.282, *p*=0.019*
Conduct problems					χ^2(3)=7.9, *p*=.048*	
Obsessive compulsive problems		*t*(41)=-3.58, *p*=0.009**	ρ=.437, *p*<0.0001***			
Trauma scales						
Hyperarousal						ρ=.261, *p*=0.03*
Intrusive				ρ=.244, *p*=0.03*		
Avoidance/Numbing	*U*=466, *Z*= -2.87, *p*=0.016*	*U*=372, *Z*= -3.58, *p*<0.0001***	ρ=.442, *p*<0.0001***			ρ=.252, *p*=0.04*
RATS Total	*U*=506, *Z*= -2.379, *p*=0.034*	*U*=429, Z= -2.88, *p*=0.008**	ρ=.377, *p*=0.001**			ρ=.239, *p*=0.048*

Note: Only results significant after correcting for multiple comparisons are displayed. All relevant corrections report the Benjamini-Hochberg Adjusted *p* values.

a Males > females; *b* **Thought**: Thailand (*n*=33) < Iraq (*n*=16), Myanmar (8), Other (*n*=14) **Withdrawn/Depressed**: Thailand < DRC (*n*=7), Iraq, (*n*=16), Other (*n*=14) ; *c* **Withdrawn/Depressed**: Karen (*n*=30) < Assyrian (*n*=16), Other (*n*=22) **Attention**: Karen (*n*=30) < Assyrian (*n*=16), Other (*n*=22) **OCD**: Karen (*n*=30) < Other (*n*=22); *d* **Withdrawn/Depressed**: Karen (*n*=30) < Other (*n*=17) **Attention**: Karen (*n*=30) < Arabic (*n*=21), Other (*n*=17) ; *e* Displaced (*n*=32) > Non-displaced (*n*=46); *f* **Rule-breaking**: Unskilled (*n*=27) > No work (*n*=21) > Own business/professional (*n*=10) **Conduct**: No work (*n*=21) > Own business/professional (*n*=10)

* Significant at the 0.05 level
** Significant at the 0.01 level
*** Significant at the 0.001 level
