# Supplementary material for: Age and environmental factors predict psychological symptoms in adolescent refugees during the initial post-resettlement phase
Source: Child Adolesc Psychiatry Ment Health. 2022 Dec 20;16:105. doi: 10.1186/s13034-022-00538-y (PMC9768994; doi:10.1186/s13034-022-00538-y)
Supplement: Supplementary file 3 — Additional file 3: Table S3. Socio-demographic and clinical predictors of YSR and RATS symptom scores. [file 13034_2022_538_MOESM3_ESM.pdf]

Table S3 Socio-demographic and clinical predictors of YSR and RATS symptom scores

Predictor variable	Adj R Squared	Statistic	Part correlation
YSR Syndrome Scales			
**Anxious/Depressed**	**.219**	***F*(2,70) = 11.10, *p* <.0001**	
Age		*t*= 2.09, *p*=.040*	.242
Number of traumatic events		*t*= 3.77, *p* <.0001***	.411
**Withdrawn/Depressed**	**.297**	***F*(5,67) = 7.10, *p* <.0001**	
Age		*t*= 1.89, *p*=.063	.187
Time in Australia		*t*= 1.37, *p*=.176	.135
Ethnicity		*t*= 1.36, *p*=.179	.134
Displaced		*t*= 2.92, *p*=.005**	.288
Number of traumatic events		*t*= 1.88, *p*=.064	.186
**Social problems**	**.15**	***F*(2,75) = 7.85, *p*=.001**	
Language (group)		*t*= 3.27, *p*=.002**	.343
Parent/s in Australia		*t*= 2.84, *p*=.005**	.303
**Thought problems**	**.109**	***F*(2,65) = 5.08, *p*=.009**	
Ethnicity		*t*= 2.37, *p*=.021*	.273
Parental depression symptoms		*t*= 1.90, *p*=.062	.219
**Attention problems**	**.068**	***F*(1,76) = 5.53, *p*=.021**	
Ethnicity		*t*= 2.35, *p*=.021*	.260
**Rule-breaking behaviour**	**.088**	***F*(2,72) = 4.59, *p*=.013**	
Sex		*t*= 1.73, *p*=.089	.191
Parental pre-migration occupation		*t*= 1.94, *p*=.056	.215
DSM-Oriented Scales			
**Depressive problems**	**.145**	***F*(3,69) = 5.07, *p*=.003**	
Age		*t*= 2.78, *p*=.007**	.303
Ethnicity		*t*= 1.40, *p*=.167	.152
Number of traumatic events		*t*= 1.17, *p*=.244*	.128
**Anxiety problems**	**.091**	***F*(2,61) = 4.15, *p*=.020**	
Parental STAR-MH screen		*t*= 2.03, *p*=.046*	.191
Number of traumatic events		*t*= 1.59, *p*=.116	.244
**Attention deficit problems**	**.078**	***F*(2,72) = 4.13, *p*=.020**	
Ethnicity		*t*= 1.85, *p*=.069	.206
Parental pre-migration occupation		*t*= 1.72, *p*=.090	.192
**Oppositional defiance problems**	**.162**	***F*(2,64) = 7.39, *p*=.001**	
Parental pre-migration occupation		*t*= 2.67, *p*=.01*	.300
Parental anxiety symptoms		*t*= 3.14, *p*=.003**	.354
**Obsessive compulsive problems**	**.280**	***F*(3,69) = 10.32, *p*<.0001**	
Age		*t*= 1.26, *p*=.211	.126
Ethnicity		*t*= 2.01, *p*=.048	.201
Number of traumatic events		*t*= 3.89, *p*<.0001***	.389
**Internalising problems**	**.175**	***F*(2,69) = 8.47, *p*=.001**	
Age		*t*= 2.27, *p*=.026*	.245
Number of traumatic events		*t*= 3.10, *p*=.003**	.331
**Externalising problems**	**.065**	***F*(1,73) = 6.16, *p*=.015**	
Parental pre-migration occupation		*t*= 2.48, *p*=.015*	.279
**Total problems**	**.125**	***F*(3,69) = 4.42, *p*=.007**	
Age		*t*= 1.98, *p*=.052	.218
Parental pre-migration occupation		*t*= 2.17, *p*=.033	.239
Number of traumatic events		*t*= 1.57 *p*=.121	.173

RATS Scales			
**Avoidance symptoms**	**.375**	***F*(2,69) = 22.29, *p* <.0001**	
Displaced		*t*= 3.35, *p*=.001**	.315
Number of traumatic events		*t*= 5.17, *p* <.0001***	.485
**Total trauma symptoms**	**.338**	***F*(3,60) = 11.70, *p* <.0001**	
Displaced		*t*= 1.84, *p*=.071	.189
Number of traumatic events		*t*= 3.94, *p* <.0001***	.404
Parental depression symptoms		*t*= 2.01, *p*=.049*	.206

* Significant at the 0.05 level

** Significant at the 0.01 level

*** Significant at the 0.001 level
